# Adsorptive removal of bulky dye molecules from water with mesoporous polyaniline-derived carbon

**DOI:** 10.3762/bjnano.11.47

**Published:** 2020-04-08

**Authors:** Hyung Jun An, Jong Min Park, Nazmul Abedin Khan, Sung Hwa Jhung

**Affiliations:** 1 Department of Chemistry and Green-Nano Materials Research Center, Kyungpook National University, Daegu 41566, Republic of Korea

**Keywords:** acid red 1, adsorption, bulky dye molecules, Janus green B, polyaniline-derived carbon, water purification

## Abstract

Polyaniline-derived carbon (PDC) was obtained via pyrolysis of polyaniline under different temperatures and applied for the purification of water contaminated with dye molecules of different sizes and charge by adsorption. With increasing pyrolysis temperature, it was found that the hydrophobicity, pore size and mesopore volume increased. A mesoporous PDC sample obtained via pyrolysis at 900 °C showed remarkable performance in the adsorption of dye molecules, irrespective of dye charge, especially in the removal of bulky dye molecules, such as acid red 1 (AR1) and Janus green B (JGB). For example, the most competitive PDC material showed a *Q*_0_ value (maximum adsorption capacity) 8.1 times that of commercial, activated carbon for AR1. The remarkable adsorption of AR1 and JGB over KOH-900 could be explained by the combined mechanisms of hydrophobic, π–π, electrostatic and van der Waals interactions.

## Introduction

Dyes have been widely used in a wide range of industries including textile, leather and paper, causing serious concern worldwide mainly because of the contamination of water resources. For example, around 700,000 tons of textile dyes are produced annually; and a considerable quantity of the produced dyes is discharged into waste water [1]. Such dyes are usually toxic or are converted into toxic substances after further treatment [1–2], and dyes discarded in waste water inevitably increase the biochemical oxygen demand (BOD) and chemical oxygen demand (COD) levels. Additionally, dyes decrease sunlight penetration through water, decreasing the natural restoration activity of rivers. Moreover, dyes in waste water are also considered problematic in the aesthetic sense, since the absorbance of dyes is usually very high (therefore, even small quantity of dyes can affect the color of the water).

The removal of dye molecules from contaminated water is very important and has been carried out via various methods such as oxidation [3–4], including advanced oxidation processing (AOP), photocatalysis [5], biological treatment, coagulation, and membrane separation [2,6–7]. However, these techniques are not very satisfactory for applications on a large scale. For example, dyes are very resistant against degradation by catalysis, with a common example given by the stable characteristics of dyes under even sunlight. Recently, adsorption has been regarded to be very effective and attractive because of its operation under mild conditions and no need of oxidant, active catalyst, and irradiation [8–9]. Therefore, adsorption with carbon nanotubes, activated carbon (AC), biomass, and metallic–organic frameworks (MOFs) has been actively studied for the removal of dye molecules from water [10–15]. However, adsorbents with high adsorption capacity, structural integrity, low cost and facile recyclability are required for the practical operations or commercial applications.

So far, the development of various adsorbents has been successful because of functional carbon materials (graphene [16] or porous carbon [17]), mesoporous materials [18] and MOFs [19–22]. For example, MOFs [23–25], carbonaceous materials (such as carbon nanotubes, graphene, biochar and activated carbon) [26] and clay [27] have been applied in adsorptive removal of contaminants of emerging concern, hazardous organics and persistent organic pollutants. Carbonaceous materials have been particularly attractive in the purification of contaminated water via adsorption because of the easy preparation of carbon materials [26], especially from waste materials [28]. Moreover, highly porous carbon materials, especially with high nitrogen content, have been produced from various precursors including organic polymers [29–33] and MOFs [34–38].

Polyaniline (PANI), prepared from aniline, is a useful polymer in various fields because of its facile synthesis, high conductivity and nitrogen content. Porous carbon materials, with high porosity and nitrogen content, have also been obtained from PANI. In other words, functional carbon, for catalysts and supercapacitors can be derived from high temperature carbonization of PANI, especially in the co-presence of activating agents such as KOH, H_3_PO_4_ or ZnCl_2_ [39]. Even though PANI-derived carbon (PDC) was used in gas-phase adsorption [40–41], it has been scarcely applied in liquid phase adsorption. Only recently we applied PDC for the possible purification of water contaminated with organics and fuel containing dibenzothiophene or dimethyldibenzothiophene [42–43]. However, further research is required to utilize the highly porous PDC materials for the purification of water contaminated with organics such as dyes.

Herein, we utilized PDC, prepared especially at high temperature, for the purification of water contaminated with dyes, such as acid red 1 (AR1), Janus green B (JGB), methyl orange (MO) and methylene blue (MB), via adsorption. AR1 is a large anionic dye which is toxic and widely applied in the paper industry [44]. Janus green B (JGB) is one of the most typical large cationic dyes that is widely used in several industries [42]. MO and MB are widely applied anionic and cationic dyes, respectively [45]. The chemical structures of the studied dyes are shown in Supporting Information File 1, Figure S1.

A PDC, obtained from PANI at 900 °C, showed remarkable performance in the adsorption of bulky dye molecules such as AR1 and JGB. For example, the PDC material developed in this work shows the highest adsorption capacity compared with any reported results, so far. Moreover, the adsorption capacity of the PDC material is more than 8 times that of a commercial, activated carbon. However, the adsorptive performance of the PDC for small dye molecules, such as MO and MB, was not very impressive, albeit quite competitive against similar reported results. The adsorption mechanisms could be suggested based on the physical properties (including hydrophobicity) of PDC materials and adsorption of AR1 and JGB under a wide range of pH values (from 2 to 12).

## Results and Discussion

### Characterization of polyaniline-derived carbon (PDC)

**Figure 1:**
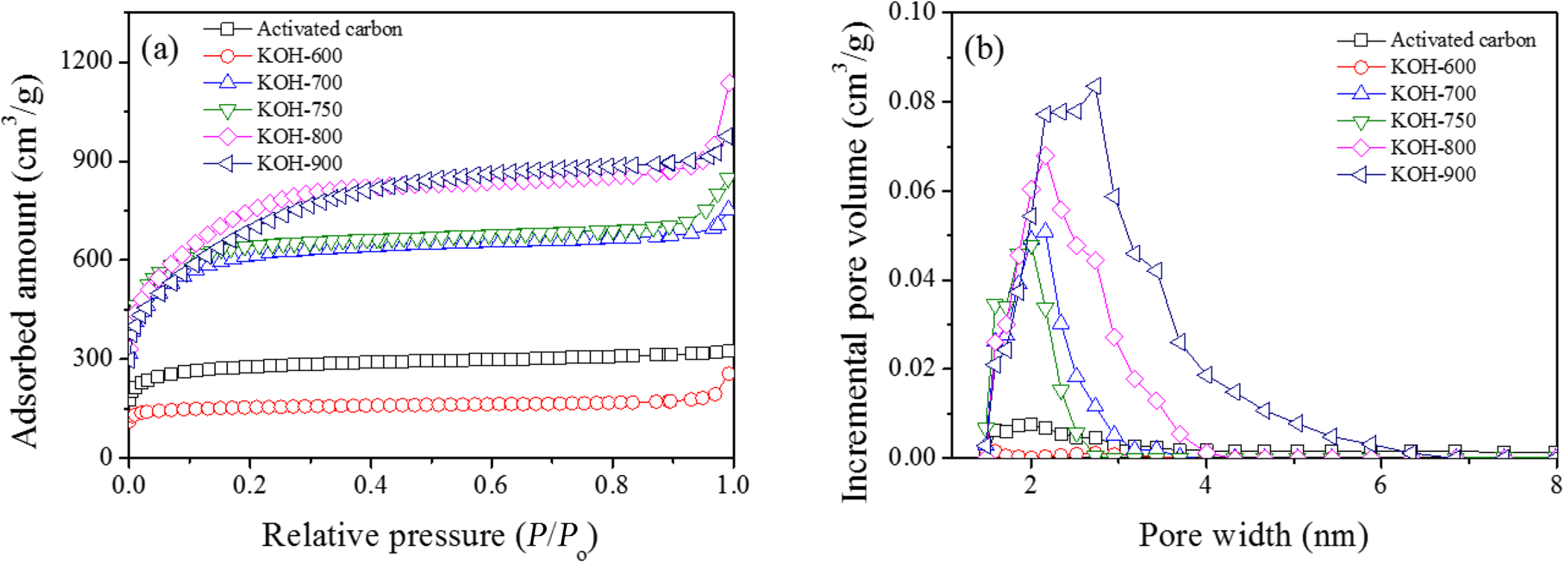
(a) N_2_ adsorption isotherms and (b) pore size distribution of PDC materials and activated carbon (AC) .

The porosity and pore size distribution of the adsorbents were characterized with nitrogen adsorption at 77 K. As shown in Figure 1a, the porosity of the PDC materials was considerable when the pyrolysis temperature was equal to or higher than 700 °C. The detailed porosity data are summarized in Supporting Information File 1, Table S1. With increasing pyrolysis temperature up to 800 °C, the BET surface area, total pore volume and mesopore volume increased. However, all of the porosity data (BET surface area, and total, micro- and mesopore volumes) decreased with further increasing temperature from 800 to 900 °C. Therefore, 800 °C was the optimum temperature to derive PDC materials with the highest porosity, excluding the micropore volume (for this, 750 °C was the most effective). Importantly, the pore size distribution patterns presented in Figure 1b show that the pore size of PDC increased with increasing pyrolysis temperature; and KOH-900, a PDC material that was obtained via pyrolysis of PANI at 900 °C, has an average pore size of ≈3 nm, which is very effective in adsorption of bulky dye molecules (vide infra). On the contrary, the pore size of activated carbon (AC) is very small (or mainly in microporous region); therefore, it might not be effective in adsorption of bulky dye molecules.

**Figure 2:**
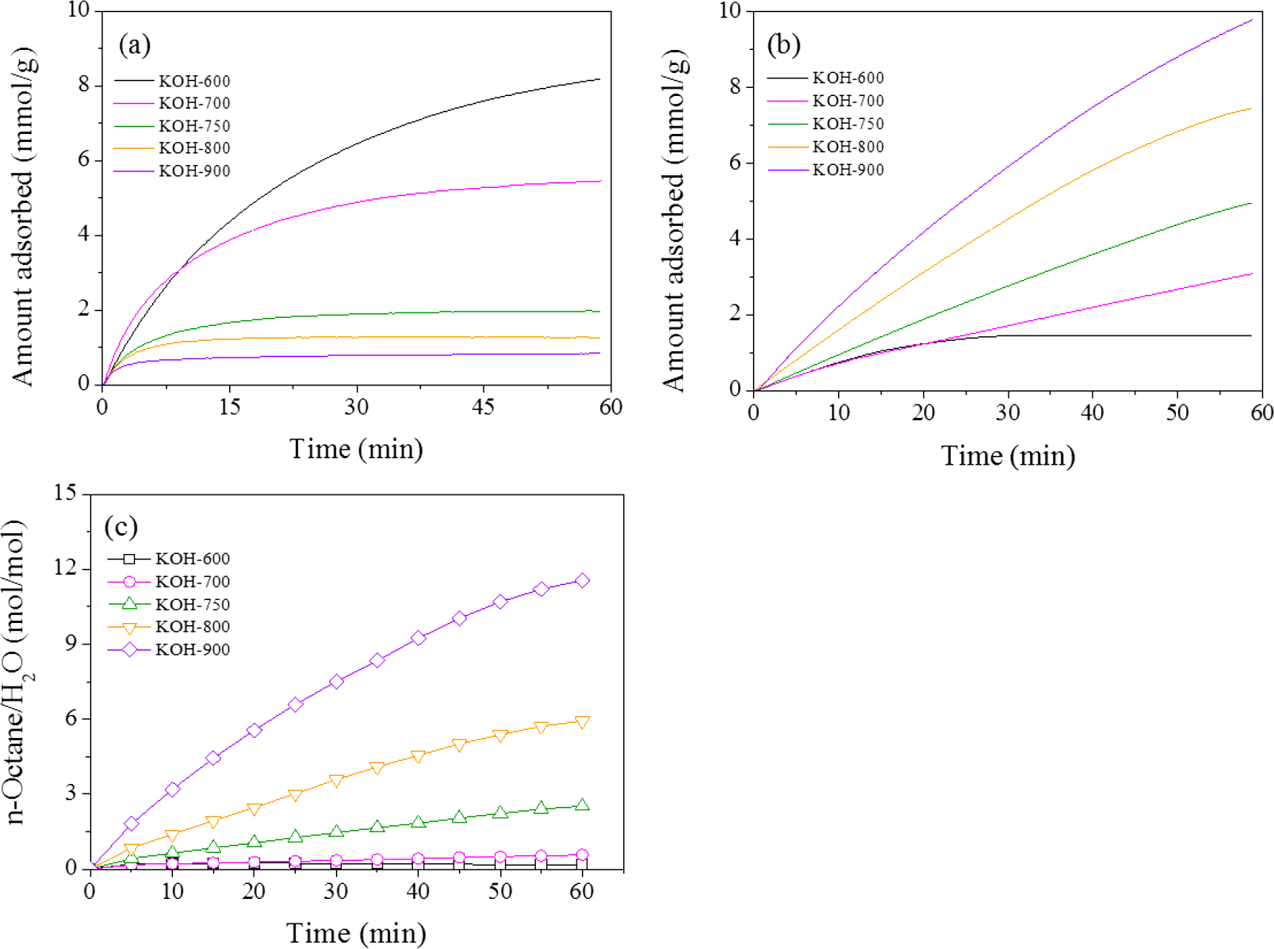
Amount of adsorbed (a) H_2_O and (b) *n*-octane over PDC materials. (c) Ratios of adsorbed amounts of *n*-octane/H_2_O over PDC materials.

The hydrophobicity or hydrophilicity of PDC was estimated by checking the adsorbed quantity of water and *n*-octane [46]. The quantity of adsorbed water decreased with increasing pyrolysis temperature, as shown in Figure 2a. On the contrary, the adsorbed *n*-octane showed the very opposite trend (Figure 2b). The adsorbed *n*-octane was in the order: KOH-900 > KOH-800 > KOH-750 > KOH-700 > KOH-600. The ratio of adsorbed vapors (*n*-octane/water, mol/mol) is shown as Figure 2c, and the ratio increased monotonically with increasing pyrolysis temperature. Therefore, it could be confirmed that the hydrophobicity of PDC increased with increasing pyrolysis temperature. This is understandable based on the enrichment of carbon (or successive removal of heteroatoms such as nitrogen and oxygen) with increasing pyrolysis temperature [42–43]. The best adsorbent (vide infra) KOH-900 was analyzed further with Raman spectroscopy. As shown in Supporting Information File 1, Figure S1, KOH-900 is composed of both graphitic and defect phases. Therefore, KOH-900 might be useful for adsorption because of defects and the graphitic layers (with π-electrons).

### Dye adsorption over polyaniline-derived carbon (PDC)

**Figure 3:**
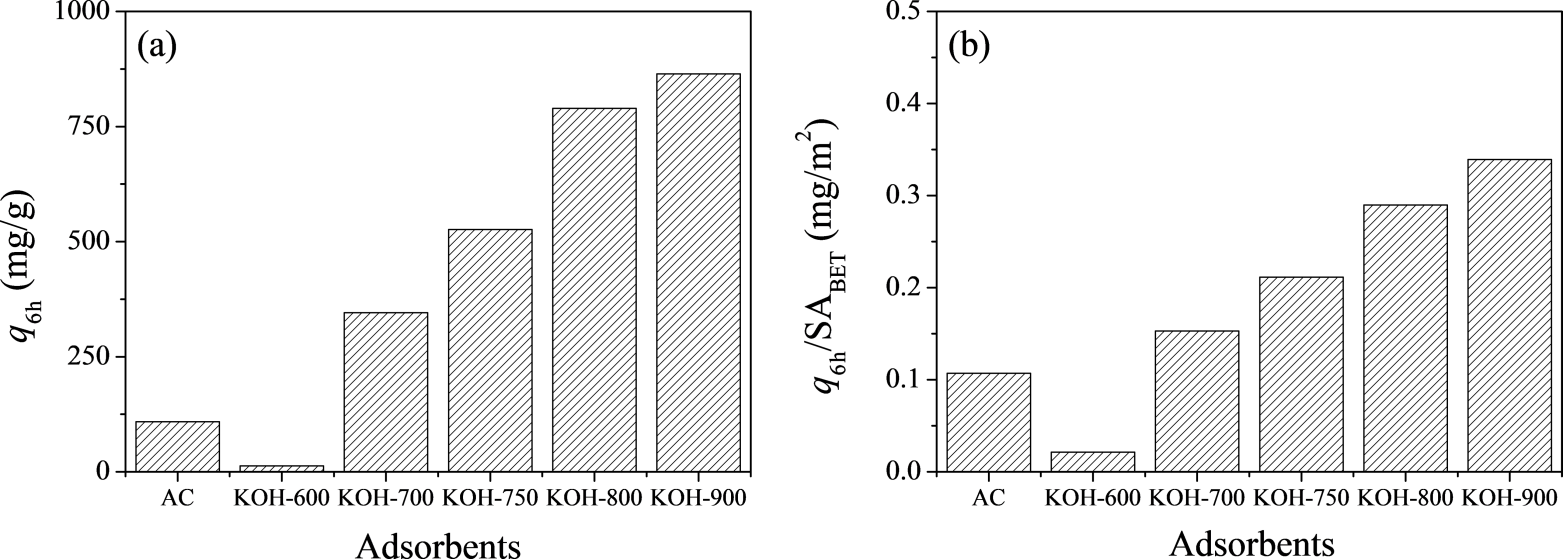
Adsorbed quantities of acid red 1 (AR1) over AC, KOH-600, KOH-700, KOH-750, KOH-800 and KOH-900 based on (a) unit weight and (b) unit BET surface area.

Firstly, adsorption of AR1 over PDC and AC was carried out for 6 h. As illustrated in Figure 3a, the adsorbed quantity (*q*_6h_, in mg/g) decreased in the order: KOH-900 > KOH-800 > KOH-750 > KOH-700 > AC > KOH-600. Therefore, the PDC materials are very competitive in AR1 adsorption compared to AC when the pyrolysis temperature is at 700 °C or higher. Considering the dominant role of porosity in adsorption [47], the *q*_6h_ values were calculated based on the BET surface area of adsorbents. Curiously, as presented in Figure 3b, the *q*_6h_ (in mg/m^2^) for AR1 showed a tendency similar to that of the quantity based on unit weight of adsorbents or PDC (*q*_6h_ in mg/g). Therefore, it should be emphasized that there is another important contribution, excluding simple porosity, to explain the performance of the PDC materials in AR1 adsorption.

**Figure 4:**
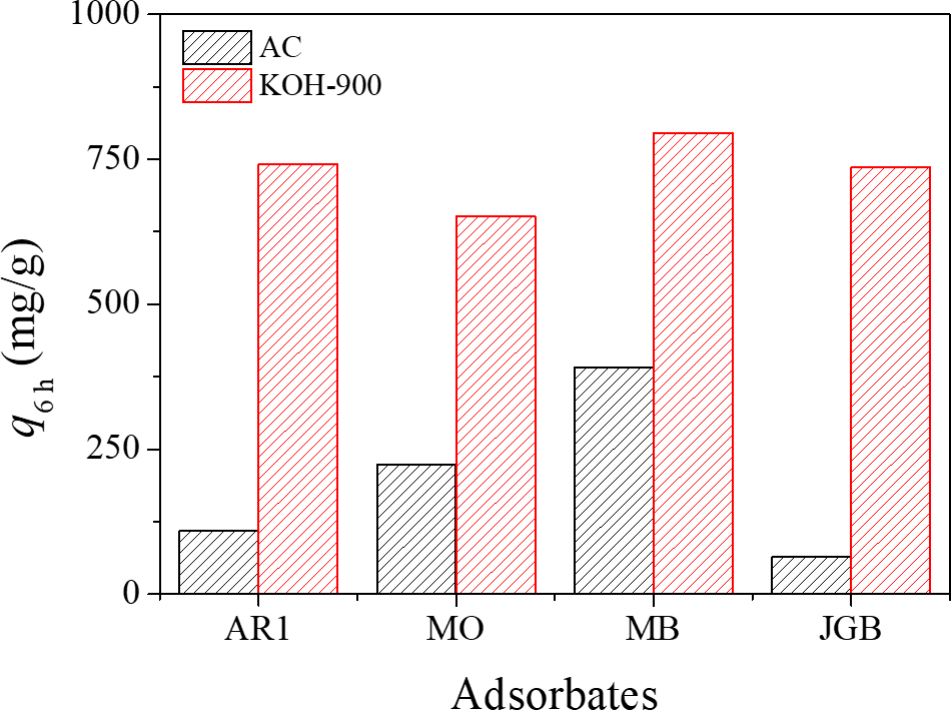
Adsorbed quantities of acid red 1 (AR1), methyl orange (MO), methylene blue (MB) and Janus green B (JGB) over AC and KOH-900.

Considering that the best performance was found with KOH-900 (based on both unit weight and BET surface area), further experiments were done with KOH-900 and AC, as a standard adsorbent. Similar to AR1, other dye molecules (with different charge and size) were also adsorbed for 6 h over KOH-900 and AC. As presented in Figure 4, KOH-900 had a much higher *q*_6h_ than AC for the four dyes studied, namely AR1, MO, MB, and JGB. However, the ratio of adsorbed quantity [*q*_6h_ (KOH-900)/*q*_6h_ (AC)] was very much dependent on the size of the adsorbed dye, as summarized in Supporting Information File 1, Table S2. KOH-900 showed much higher efficiency than AC especially in the adsorption of bulky dye molecules, such as AR1 and JGB.

**Figure 5:**
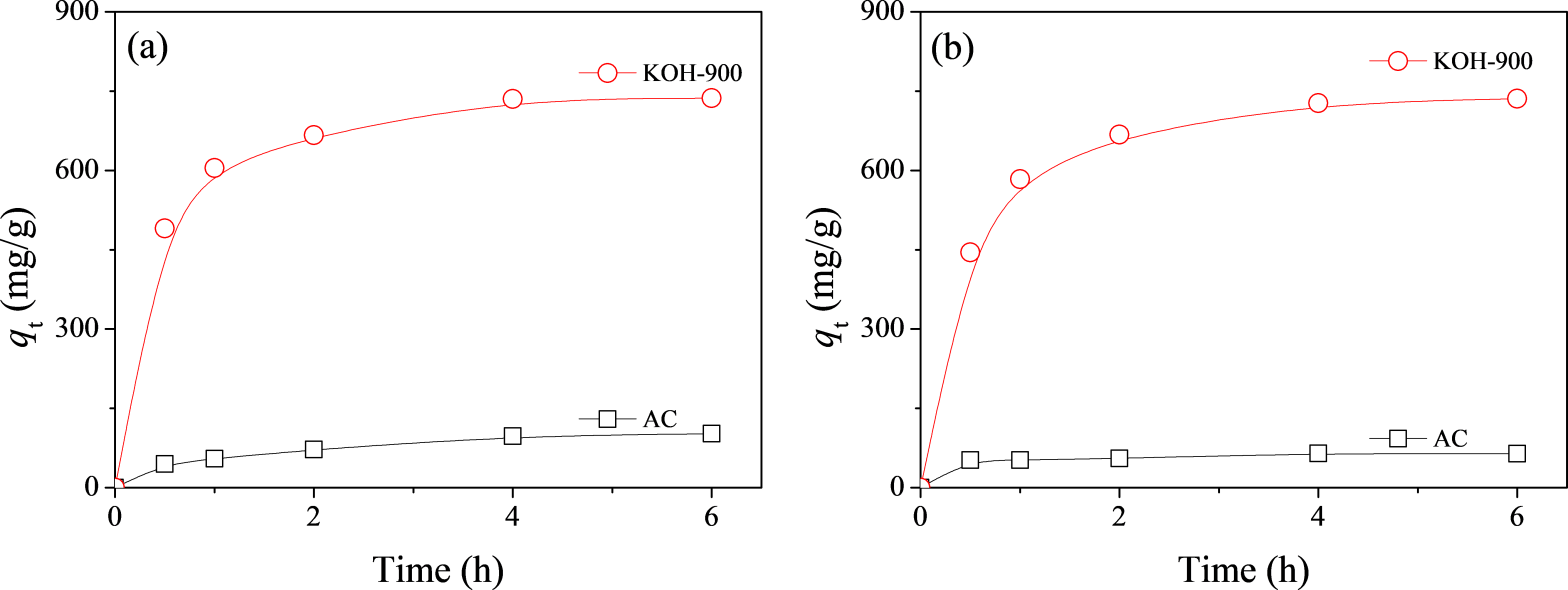
Effect of contact time on (a) AR1 and (b) JGB adsorption over AC and KOH-900.

**Figure 6:**
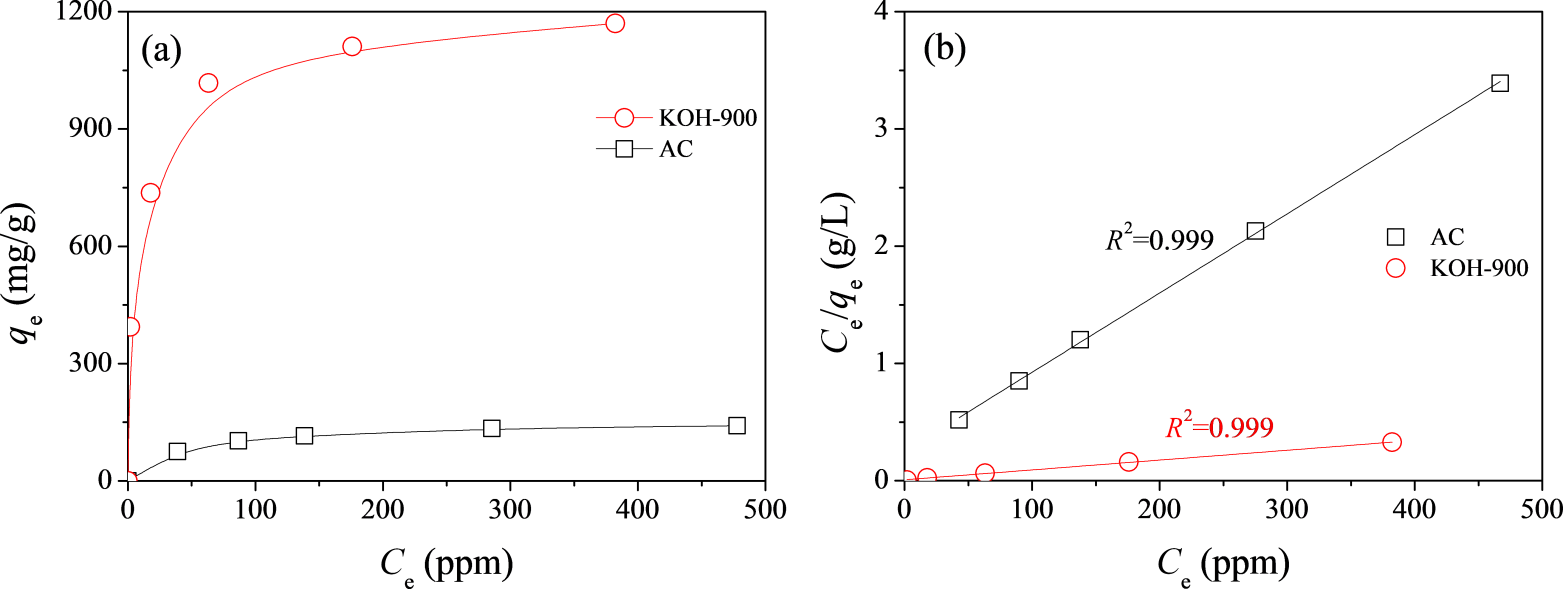
(a) Adsorption isotherms and (b) Langmuir plots for the adsorption of AR1 from water over AC and KOH-900.

Inspired by the remarkable performance of KOH-900 in the adsorption of bulky dye molecules, adsorption experiments were further carried out for AR1 and JGB over KOH-900 and AC for a wide range of adsorption times from 0.5 to 6 h. As illustrated in Figure 5, KOH-900 had a much higher adsorption capacity than AC for AR1 and JGB, irrespective of the adsorption time and the type of adsorbate or dye. In order to determine the maximum adsorption capacity of KOH-900 and AC for AR1, adsorption isotherms were obtained from adsorption for 6 h with a wide range of AR1 concentrations. The adsorption isotherms and Langmuir plots are illustrated in Figure 6a and 6b, respectively. The high correlation coefficients (*R*^2^ > 0.99) shown on Figure 6b confirm that the Langmuir equation can be adequately applied to interpret the observed adsorptions. As summarized in Table 1, KOH-900 had a *Q*_0_ (for AR1) value 8.1 times as that of commercial, activated carbon.

**Table 1:** Maximum adsorption capacity (*Q*_0_) of some reported adsorbents for the adsorption of AR1 from water.

Adsorbents	SA_BET_ (m^2^·g^−1^)	Solution pH	*Q*_0_ (mg·g^−1^)	Ref.

coal FA	9	6.0	93	[67]
Mg-Al-LDH	104	–	108	[68]
MH-1000	799	6.0	11.2	[69]
TNTs (HDTMA-modified version) treated with 0.0001 N acid	45	–	396	[70]
Fe_3_O_4_/MIL-101(Cr)	1790	5.0	143	[44]
chitosan–alunite composite	–	3.0	589	[71]
PCN-222(Fe)	2476	7.0	371	[72]
commercial activated carbon	1016	7.0	148	this work
KOH-900	2549	7.0	1192	this work

### Adsorption mechanism

Understanding the adsorption mechanism is helpful to develop a competitive adsorption technology and to further improve the performance of an adsorbent. So far, several mechanisms [48], such as electrostatic [49–50], π–π [51–54], acid–base interactions [55–56], and hydrogen bonding [57–59], were applied to interpret various adsorption events. In order to understand the plausible mechanism, especially in aqueous phase, adsorption over a wide range of pH conditions is very effective [60] since both the adsorbate and adsorbent can be changed in terms of charge or functional group (for example, via protonation or deprotonation) under different conditions of acidity/basicity.

**Figure 7:**
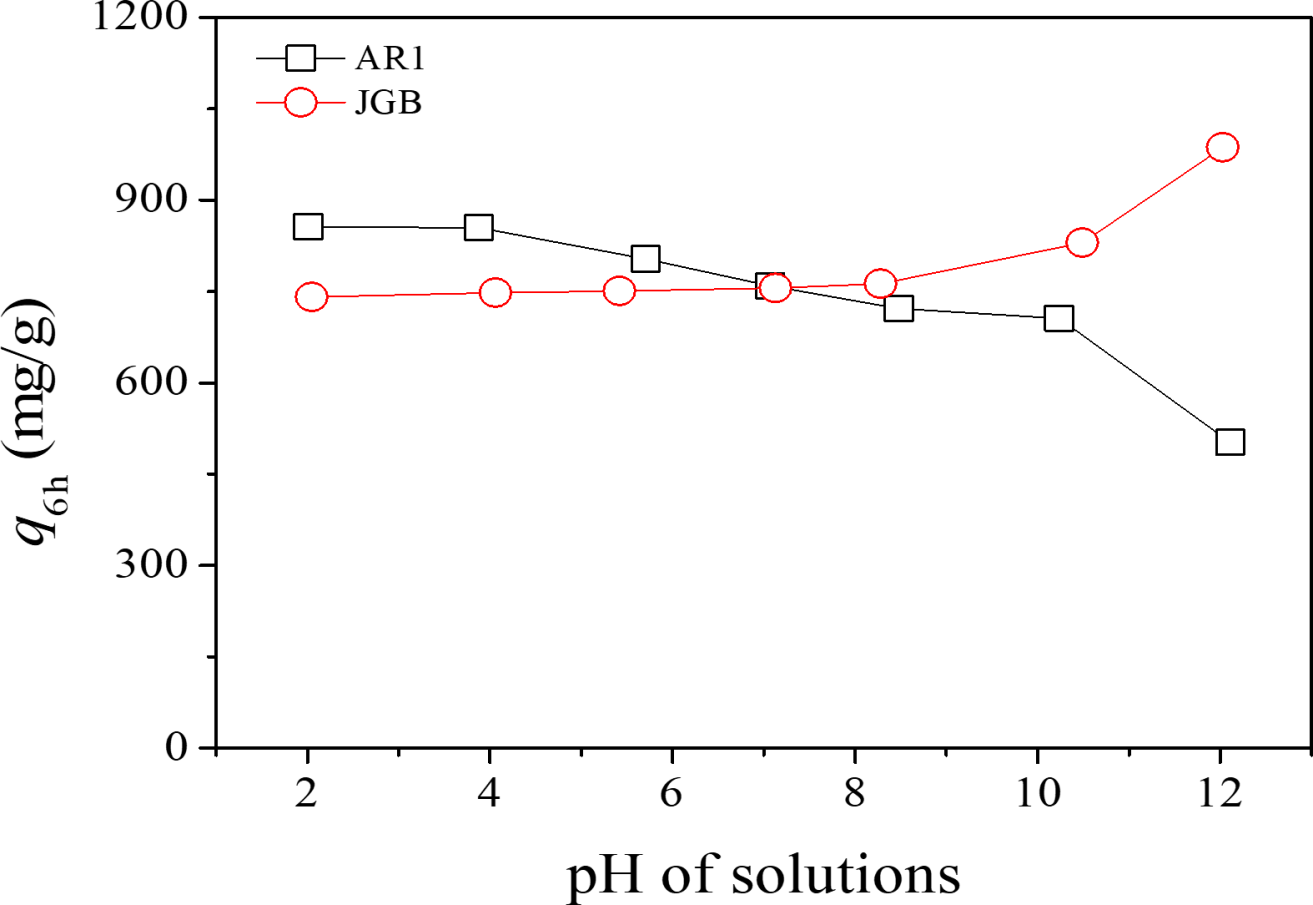
Effect of pH on the adsorbed amounts of AR1 and JGB over KOH-900.

In this study, the *q*_6h_ values were checked over KOH-900 for AR1 and JBG under pH 2–12. As shown in Figure 7, the *q*_6h_ for AR1 decreased monotonously with increasing pH of the solution; however, the adsorption of JGB showed an opposite trend. This very opposite trend, observed in anionic AR1 and cationic JGB, could be explained via electrostatic interactions considering the opposite charges on the studied dyes. Moreover, the studied PDC or KOH-900 might have both positive and negative charges, depending on pH, since the surface charge of porous carbon generally decreases (from positive to negative) with increasing pH of the adsorption solution [61–63]. Therefore, the negative AR1 should have a favorable interaction at low pH; on the contrary, the adsorption of the positive JGB will be more effective at higher pH if the electrostatic interaction is considered.

However, there should be other dominant mechanisms since the *q*_6h_ values are quite high under a wide range of pH conditions. At first, the very high hydrophobicity of KOH-900, as shown in Figure 2, can be considered. Compared with any other adsorbent, KOH-900 showed the highest performance in AR1 adsorption, as shown in Figure 3. Therefore, hydrophobic interaction can be suggested as a plausible mechanism for AR1 and JGB adsorption. This mechanism, which has been suggested earlier in adsorption of malachite green [52], aromatics [64], benzotriazole/benzimidazole [53], bisphenol A [65] and pharmaceutical and personal care products (PPCPs) [66], is acceptable considering the relatively small impact of pH on the *q*_6h_. Moreover, π–π interaction [51–54] might be another possible explanation considering that this interaction is hardly dependent on the pH (when aromatic rings are maintained under the studied pH); and both studied dyes (AR1 and JGB) and KOH-900 have ample aromatic rings with π electrons. Finally, the contribution of pore size should be mentioned. As illustrated in Figure 4 and Supporting Information File 1, Table S2, the KOH-900 sample is very effective in the adsorption of bulky dye molecules, as compared with the adsorption of small dyes such as MO and MB. This might be explained by the relatively large pore size of KOH-900, as shown in Figure 1b. Another explanation is that the pore size of KOH-900 is too large for effective adsorption of small MO or MB since van der Waals interactions rely on adequate matching between pore and adsorbates. On the contrary, bulky dye molecules such as AR1 and JGB can interact effectively with KOH-900 via van der Waals interactions, which relies on the suitable pore size of KOH-900 for the bulky dye molecules. In summary, the remarkable adsorption of AR1 and JGB over KOH-900 can be explained by the combined mechanisms of hydrophobic, π–π, electrostatic and van der Waals interactions.

### Competitiveness of KOH-900 in adsorption of dyes

Based on the remarkable performance of KOH-900 in the adsorption of AR1 and JGB, the performance of KOH-900 PDC was compared with earlier results, as shown in Table 1 [44,67–72] and Table 2 [73–77] for AR1 and JGB, respectively. As summarized in Table 1, KOH-900 had a *Q*_0_ value (for AR1) 8.1 times that of commercial, activated carbon. Moreover, KOH-900 had the highest *Q*_0_, compared with any reported adsorbent, so far. Additionally, KOH-900 showed a *Q*_0_ of more than 2 times that of a chitosan–alunite composite (previously the highest *Q*_0_) [71] even though the pH of adsorption solution was not the same. If the pH effect (vide supra, including Figure 7) is considered, the difference in *Q*_0_ between KOH-900 and chitosan–alunite composite will increase.

**Table 2:** Maximum adsorption capacity (*Q*_0_) of some reported adsorbents for the adsorption of JGB from water.

Adsorbents	SA_BET_ (m^2^·g^−1^)	Solution pH	*Q*_0_ (mg·g^−1^)	Ref.

magnetic-modified MWCNTs	145	7.0	250	[73]
ZnO/Zn(OH)_2_-NP-AC	–	7.0	98	[74]
Ni_0.5_Zn_0.5_Fe_2_O_4_	–	7.0	333	[75]
mesoporous silica	659	–	62	[76]
TiO_2_ (254 nm)	–	–	294	[77]
commercial activated carbon	1016	7.0	64^a^	this work
KOH-900	2549	7.0	736^a^	this work

^a^*q*_6h_.

Based on Table 2, KOH-900 was also very competitive in JGB adsorption against the reported adsorbents. To begin with, KOH-900 had a *q*_6h_ for JGB 11.5 times that of AC. The *q*_6h_ of KOH-900 for JGB is more than 2 times that of the *Q*_0_ of the Ni_0.5_Zn_0.5_Fe_2_O_4_ adsorbent. The difference in *Q*_0_ should increase if the actual *Q*_0_ of KOH-900 is reached (in this work, *q*_6h_ of KOH-900 was used in comparison) since *Q*_0_ is always higher than any *q*_t_ value. Therefore, it could be confirmed that KOH-900 is remarkably effective in the removal of bulky dye molecules such as AR1 and JGB, irrespective of charge, mainly because of the large pores, high porosity and high hydrophobicity.

The *Q*_0_ or *q*_t_ values of the reported adsorbents for MO and MB are compared in Supporting Information File 1, Tables S3 and S4, respectively. Even though KOH-900 is not very competitive in the adsorption of MO and MB (as compared with the adsorption of AR1 and JGB), the new adsorbent is also attractive in the removal of small dyes like MO and MB. Additionally, KOH-900 showed the second best performance in adsorption of MO or MB, partially because of its high porosity.

## Conclusion

PANI-derived carbon materials were prepared from pyrolysis of PANI under a wide range of temperatures and applied in the adsorption of dyes from water. The hydrophobicity, pore size and mesopore volume were found to increase monotonously with increasing pyrolysis temperature. In addition, the best PDC (KOH-900) was very effective in the adsorption of dyes, especially those of a large size such as AR1 and JGB. For example, KOH-900 had a *Q*_0_ (for AR1) value 8.1 times that of commercial, activated carbon. Moreover, KOH-900 showed a *Q*_0_ value of more than 2 times that of a chitosan–alunite composite which previously showed the highest *Q*_0_ to date. The remarkable adsorption of AR1 and JGB over KOH-900 could be explained with combined mechanisms such as hydrophobic, π–π, electrostatic and van der Waals interactions. Finally, the PDC materials presented in this work could be suggested as a potential adsorbent to purify water contaminated with dye molecules, irrespective of size and charge.

## Materials and Methods

### Chemicals

AR1 (60%), JGB (65%), MO (85%), MB (82%) and aniline hydrochloride (C_6_H_8_ClN, 97%) were acquired from Sigma-Aldrich. Activated carbon (2–3 mm, granule, practical grade) was obtained from Duksan Pure Chemical Co., Ltd. Other chemicals used in this research were of analytical grade and were purchased from commercial venders and applied without any purification.

### Preparation of polyaniline-derived carbon (PDC) materials

The PDC materials were obtained via pyrolysis of PANI, derived from aniline hydrochloride, in two steps, following earlier reports [42–43]. In brief, PANI was firstly pyrolyzed at 550 °C for 2 h under nitrogen flow. The pyrolyzed product was mixed well with KOH (the weight of KOH was 2 times that of the pyrolyzed product) and carbonized again at 600–900 °C for 1 h under nitrogen flow. The PDC samples were named KOH-*x* where *x* represents the pyrolysis temperature in the second step.

### Characterization of polyaniline-derived carbon (PDC) samples

PDC and AC were characterized by nitrogen adsorption (Micromeritics, Tristar II 3020) to understand their porosity characteristics. Nitrogen adsorption was carried out at 77 K after evacuation of samples at 150 °C for 12 h. The Brunauer−Emmett−Teller (BET) equation and *t*-plot were applied to calculate the surface area and micropore volume, respectively, of the adsorbents. The pore size distributions were calculated with nonlocal density functional theory (NLDFT). The hydrophobicity of the studied adsorbents was evaluated by measuring the relative quantities of adsorbed water and *n*-octane at 30 °C with thermogravimetric analysis (TGA, Perkin-Elmer TGA 4000 system), similar to a previous work [46]. In brief, an adsorbed quantity of water was measured for up to 60 min by feeding water vapor with the help of a nitrogen carrier. The adsorbed quantity of *n*-octane was determined similarly, and the relative quantity (*n*-octane/water, mol/mol) was calculated accordingly.

### Adsorption of dye molecules

The adsorption of the dye molecules was carried out with model solution at pH 7.0, considering the usual pH of rainwater and river water [78]. Detailed methods to calculate the adsorbed quantity at time *t* in h (*q*_t_) and maximum adsorption capacity (*Q*_0_) [79] are shown in Supporting Information File 1. In order to understand the adsorption mechanism, the solution pH for AR1 and JGB was controlled (up to 2–12) with aqueous solution of NaOH or HCl (0.1 M each).

## Supporting Information

File 1 Additional experimental procedure “Adsorption of dyes from water” and additional experimental results.
