# Supplementary material for: Adsorptive removal of bulky dye molecules from water with mesoporous polyaniline-derived carbon
Source: Beilstein J Nanotechnol. 2020 Apr 8;11:597–605. doi: 10.3762/bjnano.11.47 (PMC7155913; doi:10.3762/bjnano.11.47)
Supplement: File 1 — Additional experimental procedure “Adsorption of dyes from water” and additional experimental results. [file Beilstein_J_Nanotechnol-11-597-s001.pdf]

BEILSTEIN JOURNAL OF NANOTECHNOLOGY

Supporting Information

for

Adsorptive removal of bulky dye molecules from water with mesoporous polyaniline-derived carbon

Hyung Jun An, Jong Min Park, Nazmul Abedin Khan and Sung Hwa Jhung

Beilstein J. Nanotechnol. **2020,** *11,* 597–605. doi:10.3762/bjnano.11.47

Additional experimental procedure "Adsorption of dyes from water" and additional experimental results

License and Terms: This is a supporting information file under the terms of the Creative Commons Attribution License (http://creativecommons.org/licenses/by/4.0). Please note that the reuse, redistribution and reproduction in particular requires that the authors and source are credited.
The license is subject to the *Beilstein Journal of Nanotechnology* terms and conditions: (https://www.beilstein-journals.org/bjnano)

Adsorption of dyes from water

Firstly, the dye solutions with the concentration of 100 mg/L of each dye including AR1 and JGB were prepared via dissolving in deionized water (with 10% of methanol) and pH of the solutions was adjusted to 7 (considering the general pH of river water and rainwater [1]) by adding 0.1 M NaOH for batch adsorption experiments under a fixed adsorbates concentration (100 mg/L). Before adsorption, adsorbents (PDCs and activated carbon) were activated via drying for overnight at 100 °C by a vacuum pump. Dried adsorbent (3.0 mg) was added to 25 mL of aqueous solution of studied dyes and shaken using a Lab companion incubated shaker for a set time at a constant speed (250 rpm) and temperature (25 °C). The residual amount of the dyes in the separated (by filtration with a syringe filter made of hydrophobic polytetrafluoroethylene, 0.5 μm) solutions was estimated from the UV absorbance (UV-1800, Shimadzu, Japan) at wavelengths of 531, 618, 464 and 665 nm for AR1, JGB, MO, and MB, respectively. Adsorption experiments were generally performed three times, and the average values were reported in this work. The Langmuir isotherm [2] (equation is shown below) was adopted to investigate the adsorption capacity. Or, the maximum capacity of adsorption (Q_0) was calculated based on the Langmuir adsorption isotherm after adsorption for 12 h under various conditions. The adsorption isotherms for different adsorbents were plotted according to the Langmuir equation [2].

$$\frac{C_e}{q_e} = \frac{C_e}{Q_0} + \frac{1}{Q_0 b},$$

where

C_e is the equilibrium concentration of the adsorbate (mg/L)

q_e is the amount adsorbed at equilibrium (mg/g)

Q_0 is the Langmuir constant (maximum adsorption capacity, mg/g)

b is the Langmuir constant (L/mg).

Therefore, the maximum adsorption capacity, Q_0, can be obtained from the reciprocal of the slope of a plot of C_e/q_e against C_e.

Moreover, the adsorbed quantities of AR1 or JGB (from 100 mg/L solution) over the KOH-900 were also measured at a wide range of pH (2-12) after adjusting the pH with aqueous HCl (0.10 M) or NaOH (0.10 M) solution.

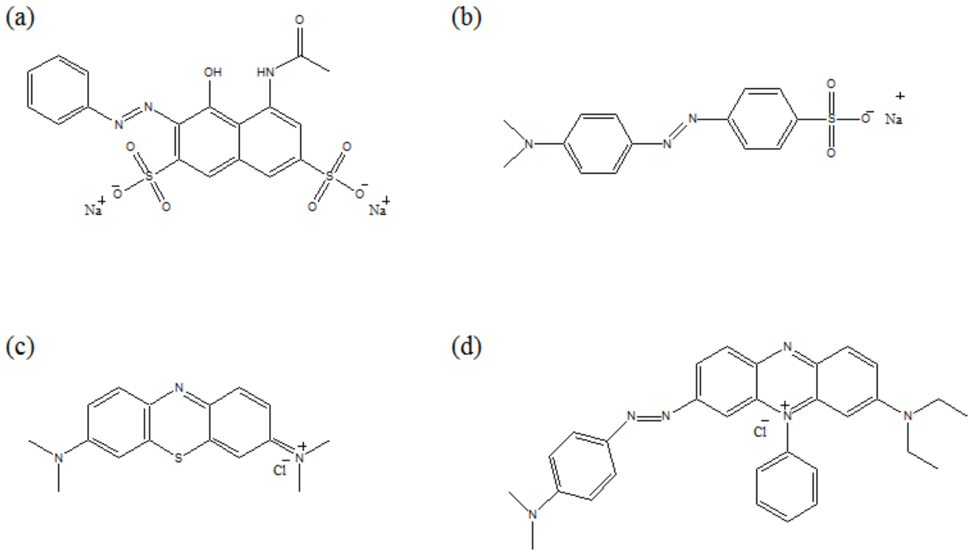

Scheme S1: Chemical structure of (a) acid red 1, (b) methyl orange, (c) methylene blue and (d) Janus green B.

Table S1: Textural properties of adsorbents.

Material	SA_{BET} (m^2/g)	PV_{total} (cm^3/g)	PV_{micro} (cm^3/g)	PV_{meso} (cm^3/g)
AC	1020	0.56	0.29	0.27
KOH-600	600	0.40	0.24	0.26
KOH-700	2260	1.17	0.97	0.20
KOH-750	2490	1.31	1.00	0.31
KOH-800	2723	1.47	0.15	1.32
KOH-900	2550	1.51	0.06	1.45

Table S2: The relative performances of KOH-900 and AC for adsorption of dyes after 6 h of adsorption.

Adsorbates	Ratio of q_{6h} (KOH-900/AC), mg/mg
AR1	6.8
MO	2.9
MB	2.0
JGB	11.5

Table S3: Maximum adsorption capacities (Q_0) of some reported adsorbents for the adsorption of MO from water.

Adsorbents	SA_{BET} ($m^2 \cdot g^{-1}$)	Solution pH	Q_0 ($mg \cdot g^{-1}$)	Reference
Mesoporous TiO_2	161	-	455	[3]
Volcanic mud	46	-	333	[4]
Phragmites australis activated carbon	1362	-	238	[5]
Activated carbon derived from finger citron residue	2887	7.0	935	[6]
PED-MIL-101	3296	5.6	194	[7]
MOF-235	-	5.6	477	[8]
Commercial activated carbon	1016	7.0	224*	This work
KOH-900	2549	7.0	652*	This work

* q_{6h}

Table S4: Maximum adsorption capacities (Q_0) of some reported adsorbents for the adsorption of MB from water.

Adsorbents	SA_{BET} ($m^2 \cdot g^{-1}$)	Solution pH	Q_0 ($mg \cdot g^{-1}$)	Reference
AC	-	-	400	[9]
Graphene oxide	32	6.0	244	[10]
SBA-15	669	-	280	[11]
Montmorillonite Clay (MC)	62	6.0	289	[12]
Poly(methacrylic acid) modified biomass	-	6.5	870	[13]
Papaya seeds	-	-	556	[14]
3D HPC	3131	-	574	[15]
MOF-235	-	5.6	187	[8]
NH_2-MIL-101(Al)	1980	-	762	[16]
Commercial activated carbon	1016	7.0	390*	This work
KOH-900	2549	7.0	795*	This work

* q_{6h}

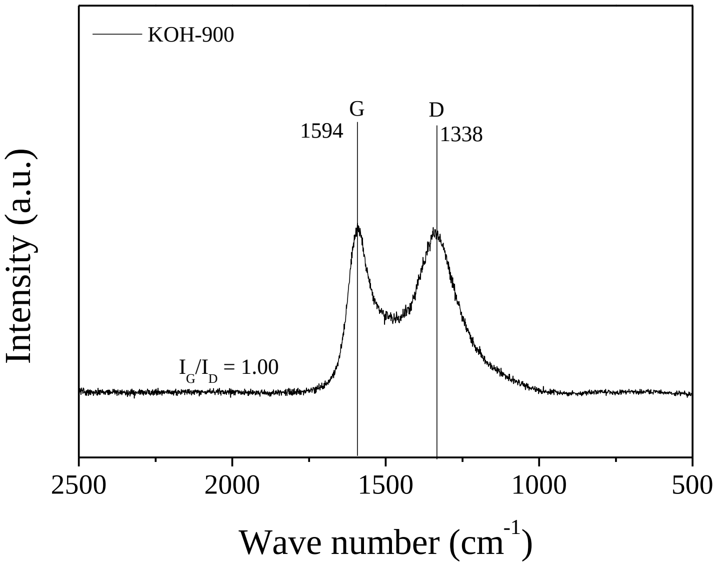

Figure S1: Raman spectrum of KOH-900.

References

1. Song, J. Y.; Bhadra, B. N.; Jhung, S. H. Micropor. Mesopor. *Mater*. **2017**, *243*, 221–228.

2. Hasan, Z.; Choi, E. J.; Jhung, S. H. *Chem. Eng. J.* **2013**, *219* 537–544.

3. Asuha, S.; Zhou, X. G.; Zhao, S. *J. Hazard. Mater*. **2010**, *181*, 204-210.

4. Jalil, A. A.; Triwahyono, S.; Adam, S. H.; Rahim N. D.; Aziz, M. A. A.; Hairom, N. H. H.; Razali N. A. M.; Abidin, M. A. Z.; Mohamadiah M. K. A. *J. Hazard. Mater*. **2010**, *181*, 755-762.

5. Chen, S.; Zhang, J.; Zhang, C.; Yue, Q.; Li, Y.; Li, C. *Desalination* **2010**, *252*, 149–156.

6. Gong, R.; Ye, J.; Dai, W.; Yan, X.; Hu, J.; Hu, X.; Li, S.; Huang, H. *Ind. Eng. Chem. Res*. **2013**, *52*, 14297−14303.

7. Haque, E.; Lee, J. E.; Jang, I. T.; Hwang, Y. K.; Chang, J.-S.; Jegal, J.; Jhung, S. H. *J. Hazard. Mater*. **2010**, *181*, 535–542.

8. Haque, E.; Jun, J. W.; Jhung, S. H. *J. Hazard. Mater*. **2011**, *185*, 507–511.

9. Kumar, K. V.; Sivanesan, S. *J. Hazard. Mater*. **2006**, *134*, 237–244.

10. Li, Y.; Du, Q.; Liu, T.; Peng, X.; Wang, J.; Sun, J.; Wang, Y.; Wu, S.; Wang, Z.; Xia, Y.; Xia, L. *Chem. Eng. Res. Des*. **2013**, *91*, 361–368.

11. Dong, Y.; Lu, B.; Zang, S.; Zhao, J.; Wang, X.; Cai, Q. *J. Chem. Technol. Biotechnol*. **2011**, *86*, 616–619.

12. Almeida, C. A. P.; Debacher, N. A.; Downs, A. J.; Cottet, L.; Mello, C. A. D. *J. Colloid Interface Sci.* **2009**, *332*, 46–53.

13. Yu, J.-X.; Li, B.-H.; Sun, X.-M.; Yuan, J.; Chi, R.-A. *J. Hazard. Mater*. **2009**, *168*, 1147–1154.

14. Hameed, B. H. *J. Hazard. Mater*. **2009**, *162*, 939–944.

15. Dai, J.; Sun, J.; Xie, A.; He, J.; Li, C.; Yan, Y. *RSC Adv*. **2016**, *6*, 3446-3457.

16. Haque. E.; Lo, V.; Minett, A. I.; Harris, T.; Church, T. L. *J. Mater. Chem. A* **2014**, *2*, 193–203.
